# Assessing the Effect of Mycotoxin Combinations: Which Mathematical Model Is (the Most) Appropriate?

**DOI:** 10.3390/toxins12030153

**Published:** 2020-02-29

**Authors:** Domagoj Kifer, Daniela Jakšić, Maja Šegvić Klarić

**Affiliations:** 1 Department of Biophysics, Faculty of Pharmacy and Biochemistry, University of Zagreb, A. Kovačića 1, Zagreb 10000, Croatia; dkifer@pharma.hr; 2 Department of Microbiology, Faculty of Pharmacy and Biochemistry, University of Zagreb, Schrottova 39, Zagreb 10000, Croatia; djaksic@pharma.hr

**Keywords:** mycotoxin interaction, Loewe additivity, combination index, isobologram, Chou-Talalay method, MixLow

## Abstract

In the past decades, many studies have examined the nature of the interaction between mycotoxins in biological models classifying interaction effects as antagonisms, additive effects, or synergisms based on a comparison of the observed effect with the expected effect of combination. Among several described mathematical models, the arithmetic definition of additivity and factorial analysis of variance were the most commonly used in mycotoxicology. These models are incorrectly based on the assumption that mycotoxin dose-effect curves are linear. More appropriate mathematical models for assessing mycotoxin interactions include Bliss independence, Loewe’s additivity law, combination index, and isobologram analysis, Chou-Talalays median-effect approach, response surface, code for the identification of synergism numerically efficient (CISNE) and MixLow method. However, it seems that neither model is ideal. This review discusses the advantages and disadvantages of these mathematical models.

## 1. Introduction

Mycotoxins are secondary metabolites mainly produced by fungi belonging to the genera of *Aspergillus*, *Penicillium*, or *Fusarium* [1]. Although the role of mycotoxins is not yet fully understood, it has been shown that mycotoxins form an integral part of microbial interactions in ecological niches where they protect fungi from competing or invading microbes (e.g., by antimicrobial activity and/or quorum sensing disruption) [2,3]. Throughout history, these fungal toxic metabolites have been recognized as harmful contaminants in crops, causing acute toxic, carcinogenic, mutagenic, teratogenic, immunotoxic, and oestrogenic effects in humans and animals [1,4]. From the public health point of view, the most important foodborne mycotoxins are aflatoxins (AFs), fumonisins (FBs), trichothecenes (including deoxynivalenol (DON) and T-2 and HT-2 toxins), ochratoxin A (OTA), patulin (PAT) and zearalenone (ZEN) and maximum levels have been set in European Union legislation to control these mycotoxin levels in food and feed [4,5]. Analytical methods based on the liquid chromatography tandem mass spectrometry (LC-MS/MS) have been developed for the simultaneous detection of multiple mycotoxins in foods which facilitated and enabled survey of their co-occurrence in various food matrices [6,7]. This methodology enabled the simultaneous detection of more than one hundred fungal metabolites including major mycotoxins as well as masked (e.g., DON-3-glucoside and ZEN-14 sulfate), modified mycotoxins (e.g., 15-acetyl-DON) and so called emerging mycotoxins (enniatins-ENN, beauvericin-BEA, and fusaproliferin-FUS and moniliformin-MON) [8,9,10,11,12,13]. The latter is defined as “mycotoxins, which are neither routinely determined, nor legislatively regulated; however, the evidence of their incidence is rapidly increasing” [13]. Recently, for the first time ever, reports were published on the multi-occurrence on major mycotoxins and their derivates as well as modified mycotoxins (such as DON-3-glucoside) and emerging mycotoxins in animal feeds and maize from Egypt. This study emphasized significant levels of AFB_1_ in this African region, but also suggested that low concentrations of the other detected mycotoxins should also be considered due to their unknown interactions [6]. As mycotoxins often co-occur in food and feed there is a possibility that, due to interactions between one or more mycotoxins, they can act harmfully, even if they are present at or below permitted concentrations (regulated mycotoxins) or are continuously present in low or high levels depending on the region (unregulated/emerging mycotoxins) [10,11,12]. Assunção et al. [5] underlined the priority of testing the most relevant mycotoxins mixtures taking into account human exposure assessments and the use of adequate mathematical approaches to evaluate interactions in experimental models. Kademi et al. [14] developed a mathematical model using a system of ordinary differential equations to describe the dynamics of AFs from plants (feeds) to animals, plants (plant foods) to humans, and animals to humans (carry-over effects) which showed that the entire dynamics depends on the numerical values of the threshold quantity defined as R_01_ and R_02_ (e.g., if R_01_ < 1 and R_02_ < 1 then AF concentrations in animals and plants will not reach toxic limit and vice versa). This kind of mathematical modeling can be useful in controlling AFs and other mycotoxin toxicity limits by employing various control measures like biological control and/or decontamination technologies. In addition, mathematical modeling has been applied to predict fungal germination, growth, mycotoxin production, inactivation and also to study the response to environmental factors which can be useful in the prediction of mycotoxin food contamination [15,16]. Taken together, mathematical modeling could be very helpful in the prediction and estimation of mycotoxin impact on human and animal health as well as in controlling contamination below acceptable limits. 

In vitro studies of mycotoxin interactions reflect mycotoxin occurrence and co-occurrence in food/feed. Among *Aspergillus*- and/or *Penicillium*-derived mycotoxins, AFB_1_, OTA, citrinin (CIT), PAT and penicillic acid (PA) have been the most studied, while the most studied mycotoxins produced by *Fusarium* species were ZEN, FBs, nivalenol (NIV), T-2, DON and its derivates. Since in the last decade attention toward unregulated/emerging mycotoxins increased, interactions of these mycotoxins as well as their interactions with major mycotoxins have also been extensively studied [17,18]. The effects of binary, tertiary and multiple mixtures of these mycotoxins in vitro have been studied on cell models originating from the digestive system, i.e., intestinal Caco-2 cells and hepatic HepG2 cells, or kidney cells like i.e., monkey kidney Vero cells, porcine PK15, human kidney HK2, and occasionally immune system-derived cells like THP-1 macrophages [18,19,20,21]. A number of studies examined the nature of interaction between mycotoxins both in vivo and in vitro classifying interaction effects into three types: antagonistic effect, additive effect, and synergistic effect [18,19]. The definition of each interaction effect is based on a comparison of observed effects with the expected effects of combination. If the observed effect is greater than expected, it is defined as a synergism, and if the opposite is true, i.e., if the observed effect is lesser than expected, it is defined as an antagonism. The third case, when the expected value is equal to the observed one is called an additive effect [22,23]. These simple definitions leave one problem though: estimations of expected effects for combinations of two non-interacting mycotoxins. Among the several available mathematical models that may be used to describe mycotoxin interactions, the arithmetic definition of additivity was the most commonly used one [24]. Other models included a factorial analysis of variance [25], Bliss independence criterion [26], Loewe’s additivity law [27], response surface [28], combination index and isobologram analysis [29], Chou-Talalay’s median effect approach [30], and the MixLow method [31]. These models will be discussed later on in this review. Additionally, the highest single agent model [32] and CISNE (code for the identification of synergism numerically efficient) [33], that have not been used so far in mycotoxicology, will also be discussed.

The most comprehensive review on mycotoxin interactions in cell cultures of human and animal origin was given by Alassane-Kpembi et al. [18]; the majority of conducted studies used the arithmetic definition of additivity. In the studies conducted in the last four years (Table 1 and Table 2) the interactions between mycotoxins in vitro were evaluated using more appropriate mathematical models than the arithmetic definition of additivity. 

## 2. Mathematical Models for Assessing Mycotoxin Interactions

In this paper, *E* will serve as an abbreviation for “effect” in equations. It is also assumed that effect is relative to maximal effect, i.e., percentage of cell viability suppression, where suppression is equal to difference between negative control (100% viability) and treated cells (100%-effect viability).

### 2.1. Simple Addition of Effects

**Figure 1:**
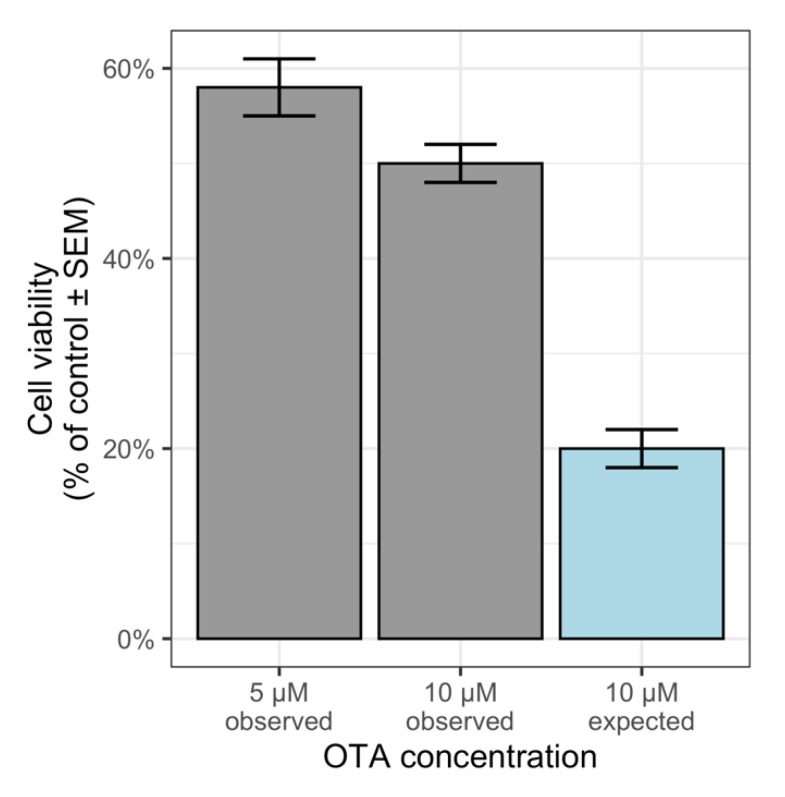
Cytotoxicity of OTA (5 μM and 10 μM observed) on PK15 cells after 24 h of exposure [34]; arithmetic additivity calculation shows that upon treatment with 5 + 5 μM of OTA expected viability is much lower than observed viability indicating antagonism (no copyright permission needed as we created this figure).

The simplest method for estimating interactions between mycotoxins is the assumption of effect additivity known as arithmetic definition of additivity or response additivity (Equation (1)):

E_exp_ = E_M1_ + E_M2_ (1)

where E_exp_ is the expected effect of combination of mycotoxin M_1_ in dose D_1_ and mycotoxin M_2_ in dose D_2_, while E_M1_ and E_M2_ are the effects of single tested mycotoxins M_1_ and M_2_ in doses D_1_ and D_2_, respectively. That simple addition of effect was applied by Šegvić Klarić et al. [34] for assessing the combination effect of beauvericin (BEA) and OTA using Equation (1) and observed synergistic effect for two combinations. Mathematically, this approach would be incorrect most of the time because the dose-effect curve is not linear. Using the data on cytotoxicity of OTA alone of the mentioned paper, it is easy to see that using this method we can prove that OTA applied in combination with itself at concentrations of 5 µM and 5 µM revealed an antagonistic effect; the expected cell viability would be around 20%, while the observed value for cell viability after treatment with 10 µM ochratoxin A was around 50% (Figure 1). Interestingly, despite an inaccurate estimation of expected effects, this model was widely applied; Alassane-Kpembi et al. [18] in their review cited 52 studies out of 83 that used this method. 

Some studies presented in Table 1 [35,36] used simple addition of effects according to Weber et al. [24] who modified Equation (1) by subtracting the 100% (or 1) from the sum of the mean effects. Needless to say, the unexplained subtraction of 100% did not account for the non-linearity of the dose response curves.

### 2.2. Factorial Analysis of Variance

This model uses simple 2-way ANOVA for modelling the detection of interactions between two mycotoxins (Equation (2)):

E = β_0_ + β_1_ × D_1_ + β_2_ × D_2_ + β_3_ × D_1_ × D_2_ (2)

where *E* is the estimated effect, β_0_ is the part of the effect achieved by negative control, β_1_/β_2_ is the coefficient that increases effect for each increase in one unit of dose D_1_/D_2_ of mycotoxin M_1_/M_2_ and β_3_ is the interaction term. 

Eight studies that have used this approach to define mycotoxin interactions were reviewed in detail by Alassane-Kpembi et al. [18]. If the interaction term was significantly (in a statistical manner) different than zero, it was concluded that an interaction between mycotoxins occurred. The main problem with this method is that ANOVA can be very misleading, similarly to the simple addition of effects method because ANOVA is based on linear modelling which is not useful for modelling nonlinear dose-effect curves [25]. This method was recently applied in only one study for testing the dual combination effects of ZEN and OTA or α-ZEL in HepG2 cells [37], as summarized in Table 1. 

### 2.3. Bliss Independence Criterion

Bliss introduced this model in 1939 for predicting the proportion of animals that will die after combining two toxins under the assumption that there is no interaction between the toxins (i.e., they have completely different mechanisms of action or act in different compartments):

E_exp_ = 1 − (1 − E_M1_) × (1 − E_M2_) = E_M1_ + E_M2_ − E_M1_ × E_M2_ (3)

where E_exp_ is the expected effect of a combination of mycotoxin M_1_ in dose D_1_ and mycotoxin M_2_ in dose D_2_, while E_M1_ and E_M2_ are the effects of single tested mycotoxins M_1_ and M_2_ in doses D_1_ and D_2_, respectively [26], all effects need to be expressed as proportions ranging from 0 to 1 (Equation (3)).

Similarly to the simple addition of effects, Bliss can result in a detection of an interaction of some mycotoxin with itself but that is not possible in model validation since this would a priori violate the assumption of two toxins acting independently.

Several of the recent studies listed in Table 1 simultaneously used different mathematical models, e.g., response additivity and Bliss independence criterion [38,39] or Bliss independence and Loewe additivity [40] or Chou-Talalay method [39,41]. As expected, these studies obtained different conclusions on mycotoxin interactions depending on the mathematical models that have been applied. For example, Smit et al. [39] obtained a synergism of DON + ZEN at low and medium concentrations by both response additivity and Bliss independence model; while at high concentrations in combinations, an additive effect was obtained with Bliss independence model and antagonism by response additivity. 

### 2.4. Loewe’s Additivity Law

Loewe’s additivity law (also called isobolografic method, concentration additivity or dose additivity) assumes that mycotoxins act within the same compartment on the same biological size by the same mechanism. The only difference is in their potency. This model is based on the dose equivalence principle and the sham combination principle; in short, every dose D_1_ of mycotoxin M_1_ gives an equal effect as D _2(1)_ of mycotoxin M_2_, and vice versa, and any D_2(1)_ can be added to any other dose of D_2_ to show the additive effect [27] as presented by Equation (4):

E (D_1_ + D_2_) = E (D_1_ + D_1(2)_) = E (D_2(1)_ +D_2_) (4)

where E is the effect, D_1_ is the dose of mycotoxin M_1_, D_2_ is the dose of mycotoxin M_2_, D_1(2)_ dose of mycotoxin M_1_ that provokes same effect as D_2_ dose of mycotoxin M_2_, D_2(1)_ dose of mycotoxin M_2_ that provokes same effect as D_1_ of mycotoxin M_1_. For additive effects, the following Equation (5) is valid:

D_1_/D_E1_ + D_2_/D_E2_ = 1, (5)

where D_1_ and D_2_ are the doses of mycotoxins M_1_ and M_2_ applied in combination, and D_E1_ and D_E2_ are the dose of mycotoxin M_1_ and M_2_ applied alone. All doses (D_1_+D_2_, D_E1_ or D_E2_) result with the same effect E.

Additionally, Loewe’s additivity law makes a larger number of assumptions; each mycotoxin in a mixture must have an equal maximum effect and all log(dose)-effect curves must be parallel and have constant relative potency [42,43], according to Equation (6):

(R = D_E1_/D_E2_) (6)

Finding two mycotoxins in a combination that fulfils all of these assumptions seems somewhat impossible. For example, apart from the Bliss independence criterion, Li et al. [44] also used this method (as a concentration addition model) to assess the nature of interaction between OTA and ZEN. Since their dose-effect curves did not meet all of the assumptions, it is easy to see that Equation (4), on which Loewe’s additivity law is based, does not hold true when we assign the values EC_10_ (OTA) = 0.8 μM and EC_10_ (ZEN) = 11.84 μM [44], and try to apply the main principles of dose equivalence and sham combination of this model (Equations (7) and (8)):

E (EC_10 OTA_ + EC _10 ZEN_) = E (EC_10 OTA_ + EC_10 OTA_) = E (EC_10 ZEN_ + EC_10 ZEN_) (7)

E (2 × 0.8 μM of OTA) = E (2 × 11.84 μM of ZEN)
(8)

This does not seem to be correct according to the dose-response curves for OTA (E (1.60 μM of OTA) ≈ 30%) and ZEN (E (23.68 μM of ZEN) ≈ 50%) presented in aforementioned article [44], which raises the question: can the observed synergies be trusted at all? 

Even though this model is mathematically valid, due to the excessive number of assumptions that need to be fulfilled, this model probably remains inapplicable for assessing combinations of mycotoxins [43]. 

### 2.5. Response Surface

Some authors expanded the Loewe’s additivity law and Bliss independence criterion to the whole surface defined by all predicted additive concentration combinations (in all ratios, for all effects) [45,46] as presented in Table 1. In mycotoxicology, Assunção et al. [46] implemented model generalization built by Jonker et al. [28]. They estimated the deviation from Loewe’s additivity law by Equation (9):

D_1_/D_E1_ + D_2_/D_E2_ = e*^G^* (9)

where G is the deviation function defined separately for 4 models. If G = 0, then Equation (9) collapses to Equation (5), suggesting an additive effect. To test for synergism or antagonism G is substituted with (Equation (10)):

G (z_1_, z_2_) = a × z_1_ × z_2_ (10)

where parameter a is less than zero for synergisms and greater than zero for antagonisms, z_1_ and z_2_ are relative contribution to toxicity, i.e., for z_1_ as presented by Equation (11):

z_1_ = D_1_/D_E1_ / (D_1_/D_E1_ + D_2_/D_E2_) (11)

Jonker et al. [28] also define more complicated interaction patterns between two toxins and with the inclusion of parameters b_1_ for detection of dose ratio-dependent deviation (Equation (12)), and parameters b_DL_ for the detection of dose level-dependent deviations (Equation (13)) from a non-interacting additive model:

G (z_1_, z_2_) = (a + b_1_ × z_1_) × z_1_ × z_2_ (12)

G (z_1_, z_2_) = a × (1 − b_DL_ × (D_1_/D_M1_ + D_2_/D_M2_)) × z_1_ × z_2_ (13)

The procedure by Jonker et al. [28] suggests fitting all four models (defined by four deviation functions) and then choosing the best one to make conclusions about the nature of the interaction at different dose ratios or dose levels based on parameters a, b_1_, and b_DL_ according to Table 1 of Jonker et al. [28].

This method provides more information than the other methods mentioned in this article, but it comes with a greater cost of the experiment since a checkerboard experimental design is needed, with dense concentration ranges in all combinations.

### 2.6. Highest Single Agent (HSA) Model

This model is also referred to as the Gaddums non-interaction [32], it defines the expected effect as the maximum of single mycotoxin effects (Equation (14)):

E_exp_ = *max* (E_M1_, E_M2_) (14)

where E_exp_ is the expected effect of a combination of mycotoxin M_1_ in dose D_1_ and mycotoxin M_2_ in dose D_2_, while E_M1_ and E_M2_ are the effects of single tested mycotoxins M_1_ and M_2_ in doses D_1_ and D_2_, respectively. 

Because of underestimations of the expected combination effect, this model is not appropriate for detection of synergistic effects, except in cases: (i) where one compound is completely inactive at any concentration for the measured effect (which is rare in the field of mycotoxins); (ii) where a mycotoxin with maximal effect does not reach full effect (i.e., never suppresses viability to 0%). On the other hand, this method is useful for detecting antagonistic effects since observing a combination effect less than the maximal effect of a mycotoxin alone clearly demonstrates an interaction of antagonistic nature. However, underestimations of the expected combination effect can hide milder antagonistic effects. The great advantage of this model is the financial cost of the experiment: to prove an antagonistic effect, it is sufficient to test three concentrations, each mycotoxin alone and a combination of the mycotoxins. Another advantage is that this method is also independent of the mechanism of action, and it does not make any assumptions on the dose-effect curve. However, this simple approach has never been applied in mycotoxicology.

### 2.7. Combination Index and Isobologram Analysis

Applying Loewe’s additivity law or similar methods can allow researchers to use the Interaction/combination index which is based on Equation (5) for describing the nature of the combination effect (Equation (15)):

CI = D_1_/D_E1_ + D_2_/D_E2_, (15)

where CI is the interaction/combination index: CI < 1 indicates synergism, CI = 1 indicates an additive effect and CI > 1 indicates an antagonism [29]. Isobologram analysis is just a “fancy” name for the graphical representation of the combination index for the same effect in different ratios of two mycotoxins. It is a simple plot with the dose/concentration of mycotoxin 1 on the x axis and the dose/concentration of mycotoxin 2 on the y axis. The characteristic line, isobole, connects the y intercept and x intercept which represents the doses needed for achieving a defined effect (i.e., 50%) for single acting mycotoxins. Plotting the dot with coordinates of doses in combination that achieve the same defined effect gives us a clue about the nature of the combination effect. All of the dots below the isobole indicate synergy, the dots above the isobole indicate antagonism, while the dots on the isobole indicate a possible additive effect [49]. The combination index and isobologram method were applied in 15 studies reviewed in Alassane-Kpembi et al. [18] and was the second most used method for assessing mycotoxin interactions and much more appropriate than the arithmetic definition of additivity or factorial design.

The problems of not meeting the assumptions of Loewe’s additivity law affect the combination index and isobologram. For example, if the two dose-response curves are not parallel, instead of one linear isobole, there will be two curvilinear isoboles around the former, linear one. The area between the two new curvilinear isoboles is not an area of synergy, nor is it an area of antagonism [43]. A recent study by Anastasiadi et al. [50] generalized the Loewe’s model accounting for nonparallel dose-response curves. As a result, Equation (15) was expanded to Equation (16):

CI = (D_1_/D_E1_)^m1/m2^ + D_2_/D_E2_, m_1_ < m_2_ (16)

where m_1_ and m_2_ are the slopes of the dose-response curves for mycotoxin 1 and mycotoxin 2.

**Figure 2:**
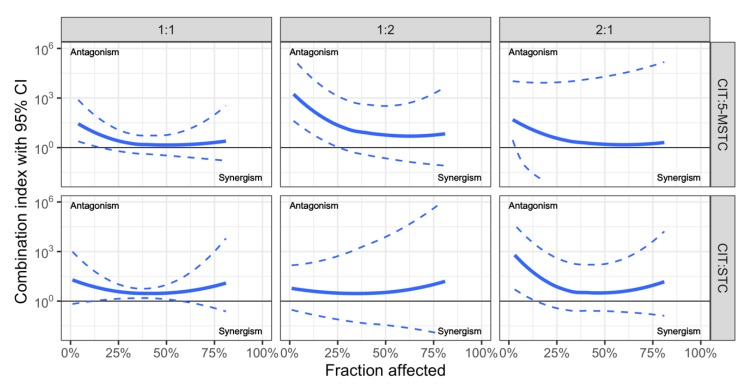
Combination indices calculated according to Anastasiadi et al. [50] accounting for different slopes of dose-response curves, 95% confidence interval (CI) was estimated using Monte Carlo simulations (N = 10000). All dose-response curves were fitted using non-linear regression. Results show mostly additive effect, with an exception of CIT + 5-M-STC combination which shows antagonistic effect in the area of up to 20% of cells affected, and CIT + STC combination (only 1 IC_50_: 1 IC_50_ ratio) in the area of 25–50% of cells affected.

Recently we tested the cytotoxicity (MTT test, 24 h) of single CIT, STC and 5-M-STC and dual combinations of CIT with STC and 5-M-STC in A549 cells (Table 2). The cytotoxicity of the mycotoxins was as follows: 5-M-STC (IC_50_ = 5.5 µM) > STC (IC_50_ = 60 µM) > CIT (IC_50_ =128 µM). Mycotoxin interactions of 1:1, 1:2 and 2:1 of IC_50_ concentration ratios were tested by applying a concentration addition model with correction for unparalleled dose-response curves as developed by Anastasiadi et al. [50], as presented in Figure 2.

### 2.8. Chou and Talalay’s Median Effect Approach

Chou and Talalay developed a unified general theory for the Michaelis-Menten, Hill, Henderson-Hasselbalch, and Scatchard equations, mathematically presented by Equation (17):

E = 1/1 + (D_M_/D)^m^ (17)

where E is the effect (between 0 and 1), D is the dose, D_M_ is the median effective dose (i.e., EC_50_) and m is a parameter for shape definition (if m < 1 dose-effect curve is hyperbolic, and if m ≥ 1 dose-effect curve is sigmoidal) [30]. Using Equation (17), it is possible to estimate the doses needed to achieve a particular effect which can be used in Equation (15) for the estimation of CI, which is then used for assessing the nature of the combination effect. Similarly to Loewe’s additivity model, the isobologram can be constructed. The Chou-Talalay model combined with an isobologram has been applied in the majority of the recently published studies [39,51,52,53,54,55,56,57,58,59,60,61,62,63,64,65] listed in Table 2. Its great advantage is the recent development of a method for the estimation of confidence intervals for the combination index which enables the application of statistics [66]. This method can easily be implemented using the web-based CalcuSyn software which automatically calculates dose-effect curves and combination indices. 

### 2.9. MixLow Method

Compared to the Chou-Talalay method, the MixLow method (Table 2) used by Lin et al. [67] improves model fitting and removes bias by fitting the log-logistic curve without prior linearization, similarly to the CISNE method (discussed in Section 2.10). However, another improvement of the MixLow method is the inclusion of random effects in a model that can account for different batches (trays) in the experiment and fit the model for both toxins and combination simultaneously [31]. Mixed modelling enables a more precise estimation of the combination index’s (CI, here called Loewe’s index) and more reliable confidence intervals or standard errors by accounting for both the error of single applied mycotoxins and combinations.

The MixLow method comes with the *mixlow* R package, which also includes functions for straigthforward data import and minimal data preprocessing, especially if the pattern suggested on the tray is followed during experimental design [68].

### 2.10. CISNE (Code for the Identification of Synergism Numerically Efficient)

Even though Chou-Talalay’s method exceeded two and a half thousand citations in relevant article databases, it does possess some technical problems in model fitting leading to bias inclusion in parameter estimation. By Chou-Talalay’s protocol Equation (17) is rearranged and transformed to linear form (Equation (18)):

log[E/(1 − E)] = m × log(D) − m × log(D_M_), (18)

where y is log[E/(1 − E)], the intercept is -m × log(D_M_), the slope is m, and x is the log(D) of the linear equation form. Estimating slope and intercept by least squares fit, and calculating D_m_ as presented by Equation (19):

D_M_ = e^−intercept/slope^ (19)

This leads to bias, along with the exclusion of data points with effects smaller than 0% or larger than 100% (i.e., stimulation) which could not be used in the logarithm on the left side of Equation (18). García-Fuente et al. [33] showed that these biases can lead to significant false positive or false negative errors, depending on the slope of the dose-response curve. They also found that fitting the same equation as a non-linear regression model estimates model parameters better and reduces the rate of false positives or negatives, especially when the slope (m) deviates from 1. This non-linear regression can be easily applied using the free CISNE software [69]. In contrast, it has not yet been applied in mycotoxicology combination testing.

### 2.11. Other methods

Most of the recent studies used mathematical modelling according to Bliss or/and Loewe (or some modified Loewe’s method) for assessing the nature of the effect of combination of mycotoxins. However several in vitro studies assed mycotoxin combined effects comparing the effect of combination to the effect of single mycotoxin [70,71,72] or only to negative controls [73,74,75,76] without estimating the theoretical (expected) effect of the combination (Table 3). Conclusions based on those studies are unreliable because the question of the nature of interaction of combination has not even been asked in a scientific manner to get a clear and exact answer. For example, Smith et al. [75] did not define the nature DON + ZEN interaction in HepRG cells; since the cytotoxic effect of a single DON was similar to the effect of DON + ZEN, it was concluded that a combined effect could not be classified as antagonistic nor synergistic. Any conclusion about an antagonistic or synergistic effect should include the effect of ZEN too, since it is a part of the mycotoxin combination.

## 3. Conclusions

Some of the methods found in studies assessing the effects of mycotoxins combination have been incorrectly based on the assumption that mycotoxin dose-effect curves are linear (simple addition of effects, factorial analysis of variance). For that reason, many conclusions have been derived incorrectly in published articles or review articles based on published data. There are many articles reviewing methods and discussing the problem of the misuse of some method, but it seems that the problem persists. The only appropriate approach to assess the nature of an interaction is to correctly estimate the dose-effect curves of each mycotoxin and combination and apply a well-defined model (based on Bliss or Loewe’s theory) with respecting the model’s assumptions and fitting the model by a direct estimation of all model parameters from a nonlinear least squares fitting. Results should be presented in a simple and clearly defined way (i.e., isobologram or combination index) with some of the most expected (mean) values accompanied by uncertainty bounds, where a 95% confidence interval should have priority over the standard error due to asymmetrical distributions. 

Improvements to the presented methods are continuously being made but are not readily applied in the field of mycotoxicology. 

## Figures and Tables

**Table 1:** Interactions between mycotoxin combinations in vitro assessed by simple addition of effects, full factorial analysis, Bliss independence criterion, Loewe additivity law and response surface.

Mycotoxin Combination	*In Vitro* Model	Mathematical Model Applied for the Endpoint	Endpoint Combined Effect	Reference
AFM_1_ + OTA	Caco-2/ human colon HT29-MTX co-cultures (100/0, 90/10, 75/25 and 0/100)	Simple addition of effects	Cell viability (Enhanced Cell Counting Kit-8, CCK-8): synergism in all cultures TEER: antagonism in all cultures, except additive effect in 90/10 co-culture Intestinal mucin *MUC2* and *MUC5B* mRNA expression: synergistic effect in 75/25 and 0/100 cultures at 4 μg/mL additive effects at the low concentration (0.05 μg/mL) culture, antagonistic effects in 100/0 and 90/10 cultures at 4 μg/mL Intestinal mucin *MUC5AC* mRNA expression: antagonistic effect in 100/0 cultures, an additive effect in 0/100 cultures at two concentrations of the mixtures Intestinal mucin MUC5AC, MUC2 AND MUC5B on protein level: synergism at 0.05 and 4 μg/mL additive effect at 0.05 μg/mL in 75/25 and 90/10 cultures	[35]
AFB_1_ + FB_1_	HepG2 cells	Simple addition of effects and factorial analysis (two-way ANOVA)	Cell cycle analysis (flow citometry assay): synergism on apoptosis at 10% and 30% of IC_50_	[36]
ZEN (30 or 60 µM) + OTA (6 or 12 µM) ZEN (30 or 60 µM) + α-ZEL (15 or 30 µM)	HepG2 cells	Full factorial analysis: 3 × 3 two-way ANOVA matrix	Cytotoxicity (MTT test): synergism of ZEN (60 µM) + α-ZEL (15 or 30 µM) antagonism in all other combinations Oxidative stress parameters (MDA, GSH, Gpx, SOD): synergism of ZEN (60 µM) + α-ZEL (15 or 30 µM) antagonism in all other combinations	[37]
DON + ZEN	Bi- and tri-culture systems: A) Caco-2 and HepaRG; B) Caco-2 and THP-1; C) HepaRG and THP-1 D) Caco-2, HepaRG and THP-1	Response additivity, CI_RA_) and Bliss independence criterion (independent joint action, CI_IjA_); IC_10_ (1:1) and IC_30_ (1:1)	Cytotoxicity (MTS test): additive effect for combination of IC_10_ in A–D (CI_RA_ and CI_IjA_) synergism for combination of IC_30_ in A–C (CI_RA_ and CI_IjA_) additive effect for combination of IC_30_ in D (CI_RA_ and CI_IjA_)	[38]
DON + MON DON + FB_1_ DON + ZEN NIV + T-2	HepaRG cells	Response additivity (CI_RA_) and Bliss independence criterion (independent joint action, CI_IjA_)	Cytotoxicity (MTS): synergism of DON + MON in all combinations except additive effect at highest concentration (1:1) (CI_RA_ and CI_IjA_) synergism of DON + FB_1_ in all combinations (CI_RA_ and CI_IjA_) except additive effect at highest concentration (1:1) (CI_RA_) synergism of DON + ZEN at low and medium concentrations (CI_RA_ and CI_IjA_); additive effect (CI_IJA_) and antagonism at high concentrations (C_RA_) NIV + T-2 synergism at low concentrations (CI_RA_ and CI_IjA_); additive effect or antagonism (CI_IJA_) and antagonism at medium and high concentrations (CI_RA_)	[39]
AFB_1_ + ZEN AFB1 + DON ZEN + DON AFB1 + ZEN + DON	HepG2 cells	Bliss independence criterion (IA) and Loewe additivity models (CA); CI-Isobologram method	Cell number (high content analysis by fluorescent labelling: IA and CA model: deviation from the obtained results; better consistency was achieved by CA model; CI model: antagonism at low fraction affected (0.05–0.15) changing to additive and synergistic effect as fraction affected increases for all combinations	[40]
TeA + ENN B; TeA + ZEN; TeA + DON; TeA + NIV; TeA + AURO; ENN B + ZEN; ENN B + DON; ENN B + NIV ENN B + AURO; ZEN + DON; ZEN + NIV; ZEN + AURO; DON + NIV; DON + AURO	Caco-2 cells	Bliss independence criterion combined with CI calculated by Chou (C) and Chou-Talalay (CT) method	Cytotxicity (WST-1 test): additive effects of binary mixtures at low concentrations calculated by Bliss independence criterion antagonism of binary mixtures ENN B, ZEN and DON as well as binary combinations of *Fusarium* toxins with TeA applied at cytotoxic concentrations as calculated by CI	[41]
ATX II + AOH	HepG2, HT29 cells and human corneal epithelial HCEC cells	Bliss independence criterion, constant ratio of 1:10 or 1:1	Cytotoxicity (WST-1 test): dominant additive effect in all cell lines antagonism in specific doses of ratios 1:10 or 1:1	[47]
AOH + DON AOH + ZEN ZEN + DON AOH + DON + ZEN	THP-1 monocytes differentiated into macrophages	Concentration addition (CA) and independent action (IA) model at equal effect concentration	CD14 expression: synergism of AOH + DON applied at low concentrations additive effects of binary and tertiary mixtures of AOH, ZEN and DON, as calculated by both CA and IA	[48]
CIT + OTA OTA + PAT OTA + MPA OTA + PA CIT + PAT CIT + MPA CIT + PA PAT + MPA PAT + PA MPA + PA	Bovine peritoneal macrophage BoMacs cells	CA and IA model; *Penicillium* toxins in IC_25_, ½ IC_25_ and ¼ IC_25_	Cell proliferation (CyQUANT^®^ GR dye): CIT + OTA synergism at ½ IC_25_ (CA, IA) OTA + PAT additive effects (CA, IA) OTA + MPA synergism at IC_25_, ½ IC_25_ and ¼ IC_25_ (CA) OTA + PA synergism at IC_25_ and ¼ IC_25_ (CA) - CIT + PAT antagonism at ½ IC_25_ (CA) CIT + MPA inconclusive (synergism CA, antagonism IA) CIT + PA antagonism at IC_25_, ½ IC_25_ (IA) PAT + MPA antagonism at IC_25_, ½ IC_25_ and ¼ IC_25_ (IA) PAT + PA synergism at ½ IC_25_; antagonism at IC_25_ MPA + PA inconclusive	[45]
OTA + PAT	Caco-2 cells	Concentration addition model (CA) and independent action (IA) model with Jonker’s generalization [28]	Cytotoxicity (MTT test): - additive effects (CA) synergism at high concentration of OTA and low of PAT (IA) antagonism at high concentration of PAT and low of OTA (IA) Gastrointestinal barrier integrity (TEER assay): synergism at low concentration and antagonism at high concentration; the change from synergism to antagonism at higher IC_50_ level (CA, IA) Genotoxicity (alkaline comet test): no dose-effect relationship of the single toxins; mathematical modelling was not applicable for the mixture	[46]

AFB_1_ and AFM_1_: aflatoxin B_1_ and M_1_, DON: deoxynivalenol, ZEN: zearalenone, α and β-ZEL: α and β-zearalenol, OTA: ochratoxin A, FB_1_: fumonisn B_1_, PAT: patulin, CIT: citrinin, MPA: mycophenolic acid, PA: penicillic acid, NIV: nivalenol, ENN A and B: enniatins A and B, AURO: aurofusarin, AOH: alternariol, ATX II: altertoxin II, TeA: tenuasoic acid, IC_10–90_: inhibitory concentration 10–90%, MDA: malondyaldehide, GSH: glutathione, Gpx: glutathione peroxidase, SOD: superoxide dismutase, MTT: (3-(4,5 dimethylthiazol-2-yl)-2,5-diphenyltetrazolium bromide) tetrazolium, TEER: transepithelial/transendothelial electrical resistance, MTS: (3-(4,5-dimethylthiazol-2-yl)-5-(3-carboxymethoxyphenyl)-2-(4-sulfophenyl)-2H tetrazolium.

**Table 2:** Interactions between mycotoxin combinations in vitro assessed by isobologram and Choul-Talalay method as well as MixLow model.

Mycotoxin Combination	*In Vitro* Model	Mathematical Model Applied for the Endpoint	Endpoint Combined Effect	Reference
AOH (50 nM-10 µM) + ZEN (10 pM-1nM) AOH (50 nM-10 µM) + α-ZEL (1 pM-1nM)	Human endometrial adenocarcinoma cell line, Ishikawa	Chou and Chou-Talalay method	Estrogenic effect (AIP test) according to C: 61% synergism, 10% additive effect, 32% antagonism for AOH + ZEN 86% synergism, 14% antagonism for AOH + α-ZEL Estrogenic effect (AIP assay) according to CT: ZEN or α-ZEL:AOH (1:250) dominant synergism Cytotoxicity (SRB assay): not possible to calculate CI	[51]
DON + T2	Human chondrocytic C28/I2, human hepatic epithelial L-02 and human tubular epithelial HK-2 cells	CI-Isobologram according to Chou-Talalay method; CI at IC_10–90_ (1:1)	Cytotoxicity (MTT test): synergism at IC_10_ in HK2 antagonism in C28/12, L-02 (IC_10–90_) and in HK2 (IC_25–90_)	[58]
DON + 15-ADON (1:1) DON + FX (3:1) DON + NIV (3:1) 15-ADON + FX (3:1) 15-ADON + NIV (3:1) FX + NIV (1:1)	Human gastric epithelial GES-1 cells	CI-Isobologram according to Chou-Talalay method; CI at IC_10_–IC_90_	Cytotoxicity (OD test): synergism of DON + 15-ADON, DON + NIV, FX + NIV at IC_10_–IC_70_; DON +FX at IC_10_ and IC_30_; 15-ADON + FX at IC_10_ additive effect of FX + NIV at IC_90_ antagonism of 15A-DON + NIV at IC_10_–IC_90_; 15-ADON + FX at IC_30_-IC_90_; DON + FX at IC_50_-IC_90_; DON +15-ADON, DON + NIV, FX + NIV at IC_90_	[59]
AFB_1_ + DON AFB_1_ + ZEN DON + ZEN AFB_1_ + DON + ZEN	HepG2 and (murine leukemia virus-induced tumor RAW 264.7 cells	CI-Isobologram according to Chou-Talalay method; CI at IC_25,50,70_ (1:1 and 1:1:1)	Cytotoxicity (Resazurin test) in HepG2: synergism of DON + ZEN AFB_1_ + DON + ZEN at IC_25–70_ additive effects of AFB_1_ + DON at IC_25–70_ antagonism of AFB_1_ + ZEN at IC_25–70_ Cytotoxicity in RAW 264.7: synergism of AFB_1_ + DON at IC_25_; DON + ZEN, AFB_1_ + DON + ZEN at IC_50,70_ additive effects of AFB_1_ + DON at IC_50,70_, DON + ZEN, AFB_1_ + DON + ZEN at IC_25_ antagonism of AFB1 + ZEN at IC_25–70_	[60]
AFM_1_ + OTA AFM_1_ + α-ZEL AFM_1_ + ZEN OTA + ZEN OTA + α-ZEL ZEN + α-ZEL AFM_1_ + OTA + α-ZEL AFM_1_ + ZEN + α-ZEL AFM_1_ + OTA + ZEN OTA + ZEN + α-ZEL AFM_1_ + OTA + α-ZEL + ZEN	Caco-2 cells	CI-Isobologram according to Chou-Talalay method; CI at IC_25,50,75,90_ (1:1, 1:1:1 and 1:1:1.1)	Cytotoxicity (MTT test): synergism of AFM_1_ + OTA at IC_50_; OTA + ZEN at IC_25,50_; OTA + α-ZEL at IC_25;_ ZEN + α-ZEL at IC_75,90_; AFM_1_ + ZEN + α-ZEL, AFM_1_ + OTA + ZEN and OTA + ZEN + α-ZEL at IC_25_; four toxins combination at IC_25,50_ additive effects of AFM_1_ + OTA at IC_25,75_; AFM_1_ + ZEN at IC_25_; OTA + ZEN and ZEN + α-ZEL at IC_50_; AFM_1_ + OTA + α-ZEL at IC_25,50_; AFM_1_ + OTA + ZEN and OTA + ZEN + α-ZEL at IC_50_ antagonism at AFM_1_ + OTA at IC_90_; AFM_1_ + α-ZEL at IC_25–90_; AFM_1_ + ZEN at IC_50-90_; OTA + ZEN at IC_75,90_; OTA + α-ZEL at IC_25_; ZEN + α-ZEL at IC_25_; AFM_1_ + OTA + α-ZEL, AFM_1_ + OTA + ZEN, OTA + ZEN + α-ZEL and AFM_1_ + OTA + α-ZEL + ZEN at IC_75,90_; AFM_1_ + ZEN + α-ZEL at IC_50-90_	[61]
ZEN + α-ZEL ZEN + ß-ZEL α-ZEL + ß-ZEL	HepG2 cells	CI-Isobologram according to Chou-Talalay method; CI at IL_25_-IL_75_ (1:1)	Cytotoxicity (NR test): synergistic effect in all combinations, except additive effect for ZEA + β ZEL at IL_25_ Expression of pro-inflammatory cytokines (IL-1ß, TNF-α, IL-8): synergism of all mixtures for IL-8 at IL_50.75_; ZEN + α-ZEL (IL_50,75_) and, ZEN + β-ZEL (IL_75_) for IL-1β and TNF-α antagonism of all mixtures for all cytokines at IL_25_ except for ZEN + α-ZEL (synergism); ZEN + β-ZEL (IL_50_) for ILβ; α-ZEL + β-ZEL for IL-1β and TNF-α at IL_50,75_	[62]
3-ADON + AOH 15-ADON + AOH 3-ADON + 15-ADON AOH + 3-ADON + 15-ADON	HepG2 cells	CI-Isobologram according to Chou-Talalay method; CI at IC_25,50,75,90_ (1:1)	Cytotoxicity (MTT test) upon 24, 48 and 72 h: dominant synergism, 3-ADON + AOH (24 and 48 h and IC_25_ 72 h), 15-ADON + AOH (24 h), 3-ADON + 15-ADON and AOH + 3-ADON + 15-ADON (all treatments) additive effect of 3-ADON + AOH IC_50-90_ (72 h); 15-ADON + AOH at IC_25,50_ (48 h) and IC_50-90_ (72 h) antagonism of 15-ADON + AOH at IC_75,90_ (48 h) and IC_25_ (72 h)	[63]
AFB_1_ + DON AFB_1_ + OTA DON+OTA	Caco-2 and HepG2 cells	CI-Isobologram according to Chou-Talalay method; CI at IC_10_–IC_90_ (1:1)	Cytotoxicity (MTT test) in Caco-2 cells: synergism of DON+OTA at IC_10_–IC_90_; AFB_1_ + DON at IC_60-90_; AFB_1_ + OTA at IC_75-90_ antagonism of AFB1 + OTA at IC_10-50_; AFB_1_ + DON at IC_10,30_ Cytotoxicity in HepG2 cells: synergism of AFB_1_ + DON at IC_10-90_; additive effects of AFB_1_ + OTA at IC_10,90_; DON + OTA at IC_60,90_ antagonism of DON + OTA at IC_10-50_	[64]
DON + PAT DON + T2 PAT + T2 DON + T2 + PAT	HepG2 cells	CI-Isobologram according to Chou-Talalay method; CI at IC_10_–IC_90_ (1:1)	Cytotoxicity (MTT test) upon 24, 48 and 72 h: no synergism dominant additive effect of DON + PAT; DON + T2 upon 72 h and at IC_75,90_ (24 h); PAT + T2 upon 72 h and at IC_10, 50-90_ (24 h); DON + T2 + PAT upon 72 h and at IC_50-90_ (24 h) and IC_25–90_ (48 h) antagonism of DON + T2 upon 48 h and at IC_10-50_ (48 h); PAT + T-2 upon 48 h and at IC_25_ (24 h); DON + T2 + PAT at IC_10,25_ (24 h) and IC_10_ (48 h)	[65]
DON + NIV (1:0.6) NIV + FX (3:1) DON + FX (1:0.2) DON + NIV + FX (1:0.6:0.2)	Jurkat human T cells	CI-Isobologram according to Chou-Talalay method; CI at IC_10_, IC_20_ and IC_30_	Cytotoxicity (MTT test): DON + NIV additive effect (IC_10_) and antagonism (IC_20,30_) NIV + FX synergism DON + FX antagonism DON + NIV + FX antagonism	[52]
DON + NIV	Differentiated three-dimensional porcine jejunal explants	CI-Isobologram according to Chou-Talalay method; CI at equimolar concentrations (1:1)	mRNA expression of cytokines: synergism in activation of all the tested pro-inflammatory genes (IL-1α,β, IL-8, IL-17A, IL-22)	[53]
DON + NIV DON + FX NIV + FX DON + NIV + FX	Human alveolar adenocarcinoma (A549) and bronchial 16HBE14o- cells primary human bronchial (hAECB) and nasal (hAECN) cells	CI-Isobologram method derived from the median-effect according to Chou at IC_10,30,50_ (1:1)	Cytotoxicity (MTT test): in A549 cells synergism of DON + NIV and DON + FX at IC_10_ and additive effect at IC_30_; antagonism of NIV + FX at IC_30_ and DON + NIV or FX at IC_50_ in 16HBE14o- cells synergism of DON + FX and NIV + FX at IC_10-50_; antagonism of DON + NIV at IC_10-50_ in hAECB cells synergism of binary mixtures at IC_10.30_ and NIV + FX at IC_50_; additive effects of DON + NIV and DON + FX at IC_50_ in hAECN cells of binary mixtures at IC_30,50_ and DON + NIV and NIV + FX at IC_10_; antagonism of DON + FX at IC_10_	[54]
DON + ZEN (1:7.5) NIV + T-2 (1:0.067) (ratio of IC_50_)	HepaRG cells	CI-Isobologram according to Chou-Talalay method	Cytotoxicity (MTS): synergism of DON + ZEN at all applied concentrations - synergism of NIV + T-2 at low concentrations antagonism of NIV + T-2 at medium concentrations	[39]
AFB_1_ + DON (1:1.44) AFB_1_ + ZEN (1:15.19) DON + ZEN (1:10.56) AFB_1_ + DON + ZEN (1:1.44:15.19)	Fibroblast cell line BF-2 from the caudal fin of *Lepomis macrochirus*	CI-Isobologram according to Chou-Talalay method; CI at IC_10_-IC_50_	Cytotoxicity (resazurin): synergism of AFB_1_ + DON and AFB_1_ + ZEN and ternary mixture at IC_10-30_ additive effect of ternary mixture at IC_40_ antagonism of DON + ZEN and ternary mixture at IC_50_	[55]
BEA + STC (1:5) BEA + PAT (3.2:1) PAT + STC + (1:5) BEA + PAT + STC (3.2:1:5)	Chinese hamster ovary CHO-K1 cells	CI-Isobologram according to Chou-Talalay method; CI at IC_5_-IC_50_	Cytotoxicity (MTT test) upon 24, 48 and 72 h: synergism of BEA + STC at IC_5,10_ (24 h); BEA + PAT at IC_5_ (24 h); PAT + STC at IC_5,10_ (24-72 h); BEA + PAT + STC at IC_5,10_ (24 h) and IC_10-50_ (72 h) additive effect of BEA + STC at IC_25,50_ (24 h), IC_50_ (48 h) and IC_10-50_ (72 h); BEA + PAT at IC_10-50_ (24 h) and IC_25,50_ (72 h); PAT + STC at IC_25_ (24-72 h) and IC_50_ (24, 48 h); BEA + PAT + STC at IC_5_ (72 h) and IC_25,50_ (24, 48h) antagonism of BEA + STC at IC_5-25_ (48 h); BEA +PAT at IC_5-50_ (48 h) and IC_5,10_ (72 h); PAT + STC at IC_50_ (72 h); BEA + PAT + STC at IC_5,10_ (48 h)	[56]
BEA + OTA	HepG2 cells	CI-Isobologram according to Chou-Talalay method; CI at IC_25_-IC_90_ (1:1) and equimolar ration (1:10)	Cytotoxicity (MTT test) upon 24, 48 and 72 h: synergism upon 72 h at IC_25_-IC_90_; 48 h at IC_25_-IC_75_; and 1:10 upon 48 and 72 h additive effects upon 24 h at IC_25_-IC_90_; 48 h at IC_90_; and 1:10 upon 24 h	[57]
CIT + STC CIT + M-STC	Human adenocarcinoma lung A549 cells	CI-Isobologram with correction for unparalleled dose-response curves, developed by Anastasiadi et al. [50]; “ray” desing with 1:1, 1:2 and 2:1 concentration ratios	Cytotoxicity (MTT test) additive effect antagonism exceptionally in low affected areas for CIT + 5-MSTC and 2:1 CIT + STC, also between IC_25_ and IC_50_ for CIT + STC	Personal unpublished data shown in Figure 2.
DON + T2	Human C-28/I2 and newborn rat primary costal chondrocytes (RC)	MixLow method; combination ratios of DON and T-2 toxin (R1=1:1 R10= 10:1, R100=100:1 and R1000=1000:1).	Cytotoxicity (MTT test): synergism at fraction affected 0.5, 0.75, 0.9 of R10 concentrations in RC antagonism at fraction affected 0.25 of R100 in both cell types; fraction affected 0.5 of R100 in C-28/12; fraction 0.5 of R1000 in RC	[67]

AFB_1_ and AFM_1_: aflatoxin B_1_ and M_1,_ DON: deoxynivalenol, ZEN: zearalenone, OTA: ochratoxin A, FB_1_: fumonisn B_1_, PAT: patulin, BEA: beauvericin, CIT: citrinin, MPA: mycophenolic acid, PA: penicillic acid, 15-ADON: 15-acetyldeoxynivalenol, FX: fusarenon-X, NIV: nivalenol, AOH: alternariol, ATX II: altertoxin II, α and β-ZEL: α and β-Zearalenol, STC: sterigmatocystin, 5-M-STC: 5-Methoxysterigmatocystin; IC_10–90_: inhibitory concentration 10–90%, CI: combination index, AIP: alkaline phosphatase, MTT: (3-(4,5-dimethylthiazol-2-yl)-2,5-diphenyltetrazolium bromide) tetrazolium, OD: optical density, SRB: sulforhodamine B assay, NR: neutral red assay.

**Table 3:** Interactions between mycotoxin combinations in vitro without applying a mathematical model.

Mycotoxin Combination	*In Vitro* Model	Statistical Analysis Applied for the Endpoint	Endpoint Combined Effect	Reference
DON + ZEN	Porcine splenic lymphocytes	ANOVA followed by the Tukey post hoc test (*p* < 0.05)	Antioxidant parameters (MDA, GSH, CAT, SOD, Gpx): synergism Apoptotic rate: synergism Expression of p53, Bcl-2, Bax, caspase-3, and caspase-8: synergism	[70]
DON + ZEN (at concentrations corresponding to the AED, TDI and ML)	HepaRG cells	Student’s t-test (*p* < 0.05)	Cytotoxicity (MTS test) upon 14, 28 and 42 days: at ML no antagonistic or synergistic effect Gene expression of CYP4F3B, CYP3A4, C/EBPα, HNF4α, aldolase B, transferrin, albumin and claudin-1 (qPCR): at AED majority of genes were ↑↑ after 14 days and ↓↓ after 28 days at TDI the gene expression upon 14 and 28 days were less different but more ↑↑ after 28 days at ML DON and DON+ZEA reduced the cell viability by more than 90%, no sufficient amounts of RNA DON + ZEN affected different genes than single DON and ZEA	[75]
DON + 3ADON (3:1) DON + 15-ADON (3:1) 3-ADON + 15-ADON (1:1) DON + 3-ADON + 15-ADON (3:1:1) (ratios of IC_50_)	HepG2 cells	ANOVA followed by the Tukey post hoc test (*p* ≤ 0.05)	Oxidative stress (ROS and MDA): binary mixtures significantly increased ROS vs. control and initial time binary and tertiary mixtures increased MDA vs. control (24, 48 and 72 h) Cell cycle distribution upon 48 h (flow cytometry): DON + 3-ADON ↓ G0/G1 and S, G0/G1 and S, G2/M phase ↑ at lower and ↓ at higher concentrations in respect to control DON + 15-ADON ↑ G0/G1 and G2/M at lower and ↓ at higher concentrations in respect to control 3-ADON + 15-ADON ↓ G0/G1 and S at all concentrations vs. control tertiary combination ↓ G0/G1, S and G2/M vs. control Micronuclei (MN): binary mixtures ↑ in MN at lower concentrations vs. control tertiary mixtures ↑ in MN at all concentrations vs. control	[73,74]
ENN A + A_1_ + B + B_1_ (1.5 or 3 µM) ENN A + A_1_ + B + B_1_ + DON (1.5 or 3 µM) BEA (2.5 µM) + DON (1.5 or 3 µM) Apicidin (0.438 µM) + DON (1.5 or 3 µM) AURO (5 µM) + DON (1.5 or 3 µM)	Porcine epithelial cells IPEC-J2	ANOVA followed by the Dunnett’s t-test or Kruskall-Wallis test (*p* < 0.05)	TEER upon 24, 48 and 72 h: dominant additive effect DON had no effect on enniatin-induced TEER decrease BEA + DON did not significantly reduce TEER	[76]
OTA + CIT	Multiple organ co-culture (IdMOC) of HepG2 and 3T3 cells	Paired sample *t*-test (*p* < 0.05)	Luciferin-IPA metabolism assay: synergism at 20% IC_50_ (CTN forms a reactive metabolite that diffuses out of HepG2 to cause cytotoxicity to 3T3 cells synergistically with OTA)	[71]
OTA + CIT (equimolar concentrations 0–30 µM)	Human embryonic kidney HEK293 cells	No statistical analysis indicated/ effect of combination was compared to the effects of mycotoxins acting alone	Cytotoxicity (MTT test): synergism based on IC_50_ of single OTA (16 µM) and CIT (189 µM) vs. combination (7 µM)	[72]

DON: deoxynivalenol, ZEN: zearalenone, BEA: beauvericin, 3-ADON: 3-acetyldeoxynivalenol, 15-ADON: 15-acetyldeoxynivalenol, ENN A and B: enniatins A and B, AURO: aurofusarin, OTA: ochratoxin A, CIT: citrinin, IC_50_: inhibitory concentration 50%, MDA: malondyaldehide, GSH: glutathione, Gpx: glutathione peroxidase, CAT: catalase, SOD: superoxide dismutase, ROS: reactive oxygen species, MTT: (3-(4,5-dimethylthiazol-2-yl)-2,5-diphenyltetrazolium bromide) tetrazolium, TEER: transepithelial/transendothelial electrical resistanceAED: average exposure dose of French adult population, TDI: Tolerable daily intake established by the JECFA, ML: maximum level permitted in cereals by the European regulation, ↑↑: up-regulated, ↓↓: down-regulated, ↑: increased, ↓: decreased.
